# R.M.O. and the War Office: Something Wrong Somewhere?

**Published:** 1915-09-25

**Authors:** 


### The Editor's Letter-Box.

### R.M.O. and the War Office Something Wrong Somewhere?

*To the Editor of* The Hospital.

Sir,—I wish to draw attention to a statement in a recent number (September 4, p. 476) where mention is made of the transference of existing staffs of infirmaries, etc., taken over by the War Office, with commissions for medical staffs—viz., the rank of "lieut.-colonel" for superintendents or heads outside the London area, and "major" for those in London, and the rank of "lieutenant" for the junior staff. That such is not the case has occurred in my own person. Our superintendent has received his "major" commisssion, but I have been left entirely out, as assistant medical officer recently appointed by the Guardians for the period of the war. There can be no real reason for such an anomaly, and the why and wherefore I am unable to divine. Anyhow, it is grossly unfair, because I am clearly entitled to a commission with higher pay; you state, in fact, that these officers have been given either the previous Guardians' salary or War Office pay, whichever may be the higher [of 24s. a day]. This is a great consideration, and a just concession for war services. Further, I ween, those over forty are not wanted for service, yet the heads are mostly very much over forty; therefore age cannot be the reason. With twenty-five years' experience, not a raw recruit just qualified, full of activity, and with no physical disqualification, one feels it would be better to start locum work, etc., at more adequate remuneration if such treatment is to prevail for the war period.

Trusting you may draw attention to such unfair discrimination.—I am, yours respectfully,

London, September 20, 1915. Peregrinus.

